# Melatonin: Manager of psychosomatic and metabolic disorders in polymorbid cardiovascular pathology

**DOI:** 10.3389/fnins.2022.989497

**Published:** 2022-09-28

**Authors:** Alexander S. Partsernyak, Victoria O. Polyakova, Artem G. Trufanov, Dmitriy S. Medvedev, Dina V. Trotsyuk, Kirill Markin, Evgeniy S. Kurasov, Evgeniya V. Kuznetsova, Alexander S. Krasichkov

**Affiliations:** ^1^Department of Military Field Therapy, Kirov Military Medical Academy, Saint Petersburg, Russia; ^2^Center for Molecular Biomedicine, St. Petersburg Research Institute of Phthisiopulmonology, Saint Petersburg, Russia; ^3^Department of Neurology, Kirov Military Medical Academy, Saint Petersburg, Russia; ^4^Department of Software Engineering and Computer Applications, Saint Petersburg Electrotechnical University “LETI”, Saint Petersburg, Russia; ^5^Department of Physiological Assessment and Medical Correction, Research Institute of Hygiene, Occupational Pathology and Human Ecology of the Federal Medical Biological Agency of Russia, Kuzmolovsky, Russia; ^6^Department of Internal Diseases, Private Educational Institution of Higher Education “St. Petersburg Medical and Social Institute”, Saint Petersburg, Russia; ^7^Department of Psychiatry, Kirov Military Medical Academy, Saint Petersburg, Russia; ^8^Department of Medical Supply, Kirov Military Medical Academy, Saint Petersburg, Russia; ^9^Department of Radio Engineering Systems, Saint Petersburg Electrotechnical University “LETI”, Saint Petersburg, Russia

**Keywords:** melatonin, circadian rhythms, metabolic syndrome, polymorbidity, cardiovascular pathology, anxiety and depressive disorders, young men, glucose

## Abstract

**Objectives:**

To investigate the relationship between changes in circadian patterns of melatonin and clinical manifestations of polymorbid cardiovascular pathology (PCVP) in young men and to analyze the effectiveness of their complex treatment.

**Materials and methods:**

We made the immunohistochemical (IHC) analysis of epiphysis tissues from autopsies of 25 men aged 32–44 with PCVP and metabolic syndrome (MS) who had died as a result of ischemic cardiomyopathy (IC) and 25 persons after the car accident as a control group. Then, 93 young men aged 35–44 with PCVP, metabolic syndrome, and depressive spectrum disorders (DSD) were divided into three groups: (1) standard therapy; (2) standard therapy and psychotherapy sessions; (3) standard therapy in combination with psychotherapeutic and psychophysiological visual and auditory correction sessions. The control group included 24 conditionally healthy male volunteers. Before and after the treatment, we studied the anthropometric status, lipid and carbohydrate metabolism indicators, the level of urinary 6-hydroxymelatonin sulfate, the degree of nocturnal decrease in blood pressure (BP), and the relationship of these indicators with circadian variations of melatonin excretion.

**Results:**

Young polymorbid patients who died from IC have a lower expression of melatonin type 1 and 2 receptors. All patients with PCVP showed a decrease in the nocturnal melatonin excretion fraction and a correlation with higher severity of depressive (*r* = −0.72) and anxiety (*r* = −0.66) symptoms. Reduced values of the 6-hydroxymelatonin sulfate (6-SM) in the 1st (*r* = 0.45), 2nd (*r* = 0.39), and 3rd (*r* = 0.51) groups before treatment was associated with periods of increased BP. The achievement of melatonin excretion reference values and normalization of biochemical parameters of carbohydrate and lipid metabolism, daily BP profile, and psychophysiological state were noted in all three patients’ groups, with a more pronounced effect in group 3.

**Conclusion:**

Low nocturnal melatonin excretion levels are associated with greater severity of clinical symptoms and a higher risk of death in patients with PCVP. Therefore, comprehensive therapy may be more effective for correcting this disease.

## Introduction

Chronodestruction of sleep, anxiety and depressive disorders, and alimentary disorders are parts of the pathogenesis of insulin resistance (IR), type 2 diabetes mellitus (DM), and obesity (Zizi et al., 2010; Lucassen et al., 2012). Sleep deprivation, combined with a shift in circadian rhythms (we mean the difference between hourly time and circadian time, for example, during shift work), is interrelated with a decrease in resting metabolic rate and an increase in glucose concentration in plasma after meals (Buxton et al., 2012). In addition, impaired sleep quality negatively affects the circadian rhythm of expression of essential metabolic modifiers (Kalsbeek et al., 2001; Laermans et al., 2015; Cui et al., 2017). The level of leptin decreases (Mullington et al., 2003), and ghrelin expression increases, which, in its turn, contributes to the feeling of hunger, despite the lower energy need for rest (Schmid et al., 2008; Beccuti and Pannain, 2011). It is important to note that the severity of jetlag is associated with obesity and metabolic syndrome (MS), and leptin resistance plays an essential role in developing these conditions (Kettner et al., 2015).

As known, food is consumed during the activity phase (daytime hours), while internal energy reserves are used during the rest phase (night time) to compensate for outlays (Gill and Panda, 2015). Consequently, the level of most metabolites, including glucose, amino acids, and lipids, varies in the blood, with maximum values during wakefulness. Since these variations are synchronous with the time of the environment, the human body uses mechanisms of absorption and release of cellular metabolites controlled by circadian biorhythms. Circadian regulation of blood glucose levels underscores the importance of circadian rhythms not only for cellular carbohydrate metabolism but also for complex mechanisms by which coordinated biorhythms of functioning of various organs, mainly the hypothalamus, liver, pancreas, and skeletal muscles, act in concert for optimal control of energy homeostasis (Van Cauter et al., 1997).

The circadian oscillator located in the suprachiasmatic nucleus of the hypothalamus controls the circadian rhythms of the body’s motor activity and the secretion of various hormones, including melatonin (MT) and insulin (Greene, 2012; Eckel-Mahan and Sassone-Corsi, 2013; Besharse and McMahon, 2016). Several studies have shown the predominant importance of the suprachiasmatic nucleus in regulating circadian variations in blood glucose levels by the phases of activity and rest. During periods of “abundance” of nutrients, myocytes, and hepatocytes absorb glucose due to insulin-dependent transporters–glucose transporters GLUT of type 2 and 4, which expression is regulated by the biological clock. The decrease in the glycemic level is compensated due to the circadian nature of glucose excretion from the liver, which depends on the daily rhythm of GLUT 2 expression, reaching its maximum during the resting–fasting phase (Dyar et al., 2014). The pancreas supports the regulation of glucose uptake and releases from tissues based on transmembrane transfer due to the rhythmic excretion of insulin and glucagon dependent on clock genes, which is accompanied by regulated rhythmic IR and the transmission of insulin signals in peripheral tissues. Thus, the feedback interaction of glucose absorption and release processes in various organs and tissues, controlled by circadian rhythms, maintains its homeostasis in the blood for 24 h (Marcheva et al., 2010; Shi et al., 2013; Zhou et al., 2014; Perelis et al., 2015).

As a result of sleep desynchronosis, the secretion of MT decreases, which is involved in the pathogenesis of obesity and type 2 DM (Espino et al., 2011; Shan et al., 2015). MT regulating circadian biorhythms also affects insulin-induced leptin expression, lipolysis, lipogenesis, and adipocyte differentiation, in which, as is known, a large number of adipokines are synthesized: adiponectin, visfatin, resistin, lipoprotein lipase, apolipoprotein E, cholesterol ester transporter protein, plasminogen activator inhibitor-1 and inflammatory interleukins (IL) 1, 6, tumor necrosis factor-α (TNF-α), etc. (Blüher and Mantzoros, 2015; Tanimura et al., 2016). These signal hormones are important factors affecting the circadian rhythm of the metabolic activity of the body, the volume (calorie) of food consumed, energy homeostasis, insulin sensitivity, carbohydrate, and fat metabolism, the state of the microcirculatory bloodstream, blood pressure (BP) and the development of metaflammation in these diseases (Nikitenko et al., 2002; Wozniak et al., 2009; Poggiogalle et al., 2018; Kuryłowicz and Koźniewski, 2020; Allada and Bass, 2021).

The combination of various diseases leads to their mutual encumbrance and the formation of polymorbidity. According to the current results of prospective studies, the prevalence of polymorbidity is up to 69% in young people, about 93% in middle-aged people, and up to 98% in elderly patients. The leading class of diseases in terms of the number of combinations formed are diseases of the circulatory system, in second place–pathology of the endocrine system, and in third place–disorders of nutrition and metabolism (GBD 2019 Diseases and Injuries Collaborators, 2020). Depressive spectrum disorders (DSD) form a significant proportion of the structure of polymorbidity and often require prescribing several groups of drugs, which, in turn, increases the risk of side effects and ineffective therapy (Pilling et al., 2009).

## Aim

Objectification of melatonin metabolism abnormalities in patients at high cardiovascular risk and receiving different therapies.

## Materials and methods

### Immunohistochemical study

In the first part of the study, we analyzed epiphysis tissue obtained from autopsy from 25 male patients aged 32–44 with PCVP and MS. In all cases, the cause of death was ischemic cardiomyopathy (IC). In addition, autopsy material of 25 persons whose cause of death was a car accident was used as a control group. All the study’s autopsy material (*n* = 50) was collected from patients whose deaths were recorded between 01.00 and 3.00 a.m. The immunohistochemical (IHC) study was performed on thin dewaxed and dehydrated sections (2–4 microns). Temperature unmasking of antigens was carried out using 0.01 M citrate buffer, pH 6.0, under pressure. A phosphate-salt buffer with Tween 20 was used for washing. The slices were incubated for 30 min with blocking serum at room temperature. After washing, primary antibodies to melatonin receptors of type 1 (Abcam 1:50) and type 2 (Abcam, 1:50) were applied. Alexa fluor 647^®^ was used as secondary antibodies. After washing, the slices were painted with DAPI, AppliChem. All samples were enclosed in a DACO mounting medium. The samples were examined using a Zeiss LM 900 confocal laser scanning microscope. The primary antibodies produced red fluorescence, and the contrasted nucleus produced blue fluorescence. In each case, 5 visual fields were analyzed at a magnification of 200 and 400 times. Fluorescence spectra were obtained to study the objects, and the relative area was determined (the ratio of all immunopositive cells to the area of all cells in the sample, %). Fluorescence with specific spectral characteristics was recorded only in the (2D) plane.

### Statistical analysis

Statistical analysis for this part of the study was carried out in Excel 2010, Microsoft Office, and the analytical program Statistica 10.0. Descriptive statistics included the calculation of medians and quartiles (since the distribution is not normal). Due to the small sample and the absence of a normal distribution, the Mann–Whitney *U*-test was used, with the help of which samples of indicators of relative expression area and optical density for all subgroups were compared in pairs with the Bonferroni correction.

### Clinical study

All participants received a complete description of the research and gave their informed consent in writing. The protocol was approved by the Institutional Review Board/Independent Ethics Committee (IRB/IEC) and conformed to ethical standards and principles described in the Helsinki Declaration (World Medical Association, 2013).

The inclusion criteria were: male patients; aged 35–44 years; established diagnosis of coronary heart disease, stage I arterial hypertension, MS, DSD. Before being divided into groups, patients had not previously received lipid-lowering statin therapy.

Exclusion criteria: any chronic diseases in the acute phase; diabetes type 1 or type 2; thyroid, central nervous system, gastrointestinal diseases; infectious, autoimmune, oncological, and hematological diseases; severe mental disorders.

The diagnosis of MS was based on 1 main and 2 additional criteria for MS. The main criteria: central (abdominal) type of obesity, waist circumference (WC) of more than 94 cm; the additional criteria: BP > 140 and 90 mm Hg. Triglyceride elevation ≥1.7 mmol/l; decreased HDL cholesterol <1.0 mmol/l; increased LDL cholesterol >3.0 mmol/l; NTG–increase in plasma glucose level in 2 h after 75 g glucose load (oral glucose tolerance test) in the range ≥7.8 and <11.1 mmol/L, with fasting plasma glucose level less than 7.0 mmol/l, IUGS–increase in fasting plasma glucose in the range ≥6.1 and <7.0 mmol/l, provided that plasma glucose after an oral glucose tolerance test is less than 7.8 mmol/l.

The diagnosis of DSD was based on a set of objective anamnesis data and separation points of The Hospital Anxiety and Depression Scale (HADS) and the depression scale of the American Center for Epidemiological Research (CES-D) that could help provide a delimitation of patients with depressive and anxiety from other patients: CES-D >18, HADS >8 (Vilagut et al., 2016; Wu et al., 2021).

All patients included in the study were divided into three equal groups by simple random selection (multiple replications where necessary). In addition, 24 healthy volunteers were selected as a control group.

Standard therapy of coronary heart disease (CHD) and arterial hypertension (AH) included: regimen, a diet with a reduced intake of fat and carbohydrates, limiting salt and fluids, metabolic therapy (trimetazidine); antianginal therapy: nitrates (isosorbide mononitrate), calcium channel blockers (verapamil, amlodipine); hypotensive drugs: angiotensin-converting enzyme inhibitors (fosinopril, perindopril) or angiotensin II receptor antagonists (losartan, valsartan), diuretics (indapamide), b-blockers (bisoprolol, metoprolol); antithrombotic drugs: antiplatelet agents (acetylsalicylic acid, clopidogrel).

Standard therapy for anxiety/depressive type DSD included: tranquilizers (tofizopam); antidepressants (serotonin reuptake inhibitors–fluoxetine, escitalopram).

Sessions of psychotherapy, including rational psychotherapy, behavioral techniques with biofeedback, relaxation techniques, and autogenic training, were conducted by a psychiatrist. A psychotherapeutic and psychophysiological visual and auditory correction was performed with a psychiatrist for 30 min, a 10-day course of 1 session per day. Visual and auditory correction is a complex correction of mental status using color and form effects from the computer monitor screen and sound accompaniment. Visual stimulation uses a frequency of 0.5–50 Hz with the demonstration of works of art, landscapes, and symbolic images. Auditory stimulation uses a frequency of 50–2000 Hz (60–400 Hz on average), with sinusoidal, triangular, sawtooth, as well as white and pink noises, which have been shown to be significantly effective in regulating consciousness and emotions (Garcia-Argibay et al., 2019). This technique aims to optimize the effectiveness of treatment of patients with mono- and polymorbid pathology occurring against the background of DSD. The main objective of visual and auditory correction is to optimize the functioning of effector systems (cardiovascular system, respiratory, and gastrointestinal systems) through changes in the main regulatory systems (immune, central nervous, and autonomic nervous systems). This method can be used at the outpatient and hospital stages. The software package used for the directed correction of the psycho-emotional state “Visual and auditory correction” has a certificate of conformity No. POCC RU.ЖТК1.H00010 dated 29.11.2018.

All patients underwent basic laboratory tests, including a general blood test and a general urine test, a biochemical study of glucose, creatinine, urea, total protein, cholesterol, low-density lipoprotein cholesterol (LDL cholesterol), triglycerides (TG) levels. In addition, the concentration of 6-hydroxymelatonin sulfate (6-SM), the main metabolite of MT, in the day and night portions of urine was determined by enzyme immunoassay. Instrumental studies were also carried out: measurement of BP by the Korotkov method, electrocardiography (ECG) in dynamics, echocardiography (EchoCG), treadmill test according to the standard protocol, daily monitoring of blood pressure (DMBP) with an assessment of the variability of the day (D) and night (N) BP (standard deviation–STD), assessment of variability and degree of reduction of night pressure (RNP). The values of the daily index distinguished the main types of circadian BP rhythm: D (dippers), systolic blood pressure (SBP), and diastolic blood pressure (DBP)–the standard (optimal) degree of night decrease in BP–10% < daily blood pressure index < 20%; ND (non-dippers) SBP, DBP–insufficient degree of night-time decrease in BP 0 < daily index < 10%; NP (night–peaker) SBP, DBP–a steady increase in night-time BP (hypertension at night) daily index <0; excessive decrease in BP at night more than 20%–“over-dipper.”

### Statistical analysis

Statistical analysis and description of the study results were carried out by the principles and requirements for conducting biomedical research. Mathematical processing was carried out using the Statistica 11.0 program. When comparing groups, the Student’s *t*-test was used for samples with a normal distribution, the Mann–Whitney *U*-test was used for non-parametric samples, and the Wilcoxon *W*-test was used to compare paired related groups if the distribution of indicators in at least one group differed from the normal one. The normality of the distribution was assessed using the Kolmogorov–Smirnov test. Spearman’s rank correlation coefficient was used to evaluate the correlation.

## Results

### Immunohistochemical study

**FIGURE 1:**
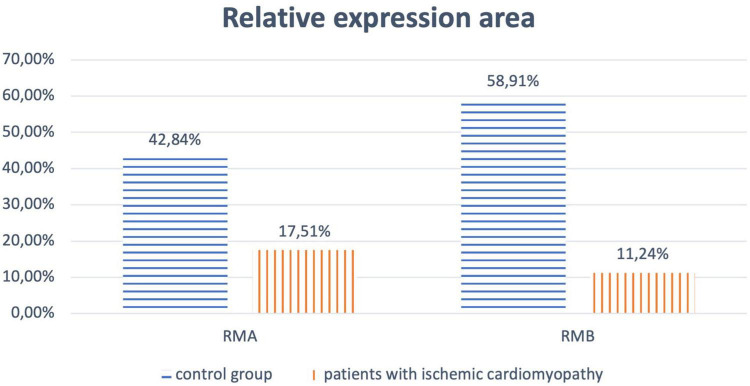
Relative expression area of melatonin receptors of type 1 (RMA) and type 2 (RMB) for patients of the main and control groups.

**FIGURE 2:**
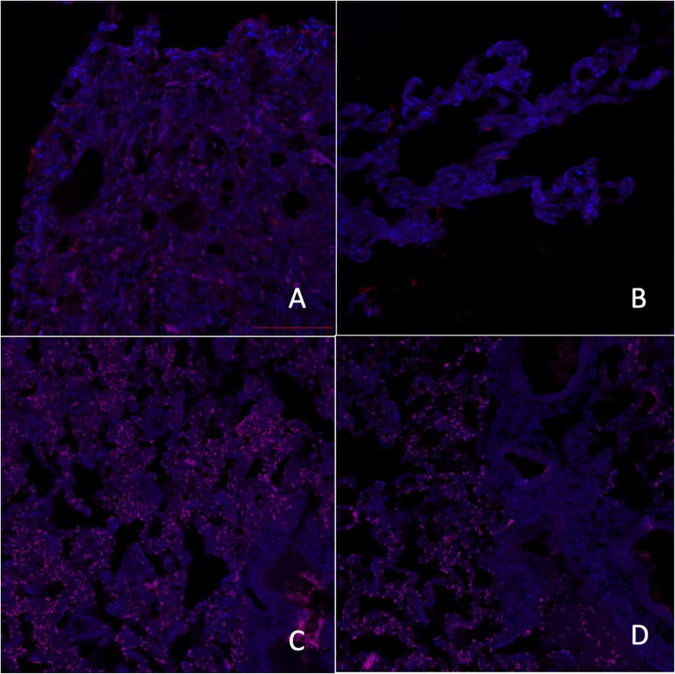
Micrographs of laser scanning confocal microscopy expression of melatonin A receptors [**A**–control group, **C**–group with ischemic cardiomyopathy (IC)] and melatonin B receptors (**B**–control group, **D**–group with IC), red fluorescence–melatonin A and B receptors, 200×.

A comparative analysis of the results of microscopy of biopsies of epiphysis tissue revealed that in patients of the main group, the relative expression area of melatonin type 1 (RMA) and type 2 (RMB) receptors was significantly lower compared to samples from the control group (*p* < 0.017) (Figures 1, 2).

### Clinical study

A total of 273 patients were recruited during the follow-up period, of whom 117 young men were included in the study: 93 male patients [age 35.0–44.0 years, body mass index (BMI) 32.0–37.0 kg/m^2^] with PCVP [CHD, functional class (FC) I-II angina pectoris, AH grade 1–3, MS, anxiety/depressive type DSD] and 24 practically well volunteers (men, average age 35.0–43.0 years, BMI up to 24.9 kg/m^2^).

As a result of the selection of patients, three representative and comparable groups with different treatment options were formed. Group 1 (*n* = 31)–male patients, average age 41.6–42.8 years, BMI 34.8–35.8 kg/m^2^, with PCVP (coronary artery disease, grade 1–3 hypertension), MS, DSD, who underwent standard therapy; group 2 (*n* = 31)–male patients, average age 42.4–43.8 years, BMI 35.4–36.2 kg/m^2^, with PCVP (coronary artery disease, grade 1–3 hypertension), MS, DSD, who underwent standard therapy and psychotherapy sessions; group 3 (*n* = 31)–male patients, average age 41.7–43.6 years, BMI 35.2–36.1 kg/m^2^, with PCVP (coronary artery disease, grade 1–3 hypertension), MS, DSD, who underwent standard therapy in combination with sessions of psychotherapeutic and psychophysiological visual and auditory correction; control group (*n* = 24)–practically well volunteers, average age 38.9 ± 2.71 years, BMI 22.91 ± 2.23 kg/m^2^, who underwent preventive medical examination. All patients with polymorbid pathology showed a shift in circadian rhythms of sleep and wakefulness to later hours, i.e., later falling asleep (more often after 24.00) and early awakening (between 06.30 and 7.30), as well as nocturnal awakenings. There were no patients with obstructive sleep apnea syndrome or restless legs syndrome among the study groups.

**FIGURE 3:**
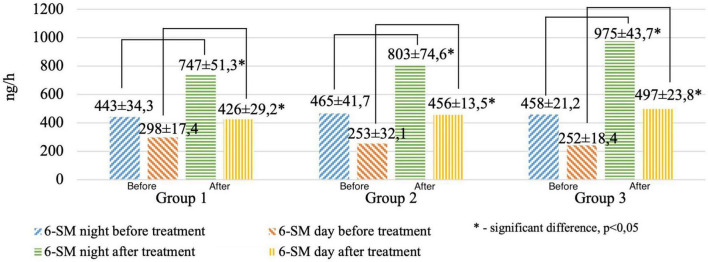
Circadian excretion of urinary 6-hydroxymelatonin sulfate before and after treatment in the study groups.

We revealed significant differences before and after treatment when evaluating the daily excretion of 6-SM (night and daytime hours) in the study groups (Figure 3).

It was found that after treatment, the patients of the 1st group had the lowest, and the patients of the 3rd group had the highest values of the 6-SM night index (the average value for the samples was 747, 803, and 975 ng/ml, respectively, *p* < 0.01), 6-SM daily index (average value for samples of 426, 456, and 497 ng/ml, respectively, *p* < 0.01). MT excretion in healthy patients averaged 399 ± 267 ng/ml during the day and 842 ± 453 ng/ml at night.

According to the data of the correlation analysis, a direct relationship was revealed between the reduced values of 6-SM in the 1st (*r* = 0.45), 2nd (*r* = 0.39), and 3rd (*r* = 0.51) groups before treatment and periods of increased systolic/diastolic BP during the 24 h. Furthermore, an increase in SBP/DBP time indices was found in the morning (06.00–08.00) and at night (03.00–06.00). After treatment, we found normal circadian variations of SBP/DBP in the studied groups, typical for polymorbid patients (Alonso-Vale et al., 2008; Espino et al., 2011).

In all groups after treatment, a modification from the “non-dipper” pattern to “dipper” and an almost complete absence of the dynamics of the “night-peaker” pattern was found (Table 1).

**TABLE 1:** Comparative characteristics of patient groups according to daily monitoring of blood pressure (DMBP) data before and after treatment (Student’s *t*-test).

Nighttime blood pressure, %	Number of cases, %							Statistical data		
		
	Group 1		Group 2		Group 3		Control group	p_1–2_	p_2–3_	p_1–3_
							
	Before treatment	After treatment	Before treatment	After treatment	Before treatment	After treatment				
Dipper	6.4	30.7	5.5	49.1	7.9	69.7	100	0.05	0.05	0.05
Night dipper	69.7	56.1	73.4	37.7	74.2	20.9	–	0.05	0.05	0.05
Night peaker	23.9	13.2	21.1	13.2	17.9	9.4	–	0.05	0.05	0.05

The *p*-value is presented with the revealed statistical significance of the paired difference between the groups.

**FIGURE 4:**
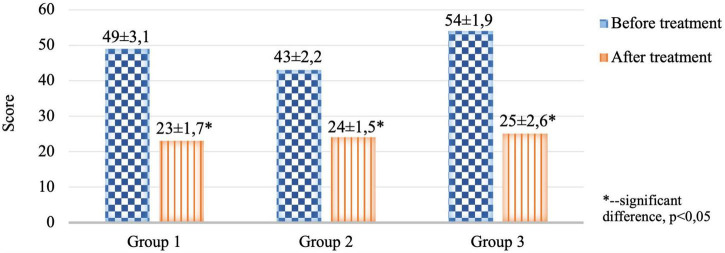
Comparative characteristics of the results of the CES-D clinical scale in the study groups before and after therapy (score).

**FIGURE 5:**
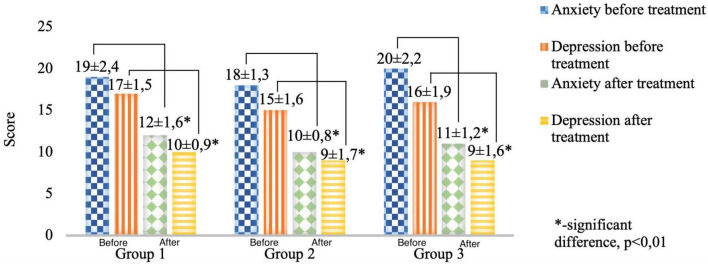
Comparative characteristics of the results of the HADS clinical scale in the study groups before and after therapy (score).

The severity of anxiety and depressive symptoms in the structure of anxiety-depressive spectrum disorders in patients with PCVP were diagnosed according to the CES-D and HADS scales (Figures 4, 5).

The severity of anxiety-depressive disorders assessed by the results of the CES-D and HADS scales in the 1st, 2nd, and 3rd groups before treatment was without intergroup differences.

When analyzing the indicators of metabolic status, attention was drawn to a statistically significant change for the better of the studied indicators in the group of patients where standard therapy was performed in combination with psychophysiological and psychotherapeutic visual and auditory correction compared with other groups (Table 2).

**TABLE 2:** Comparative characteristics of patient groups according to anthropometric parameters and the results of the examination of carbohydrate and lipid metabolism after treatment (Student’s *t*-test).

Parameter	Number of cases, %						
	
	Group 1		Group 2		Group 3		Control group
				
	Before treatment	After treatment	Before treatment	After treatment	Before treatment	After treatment	
BMI, kg/m^2^	35.5 (2.1)	34.1 (2.2)	35.8 (1.8)	33.7 (2.1)	35.6 (1.9)	31.4 (1.9)Δ	22.8 (0.9)
Waist circumference, cm	107.1 (6.3)	106.4 (5.8)	107.5 (5)	104.9 (4.4)#	107.4 (5.3)	103.3 (4.2)Δ	86 (3.9)
Glucose, mmol/l	6.5 (0.2)	5.9 (0.5)	6.5 (0.3)	5.5 (0.2)	6.6 (0.2)	5.2 (0.2) Δ	5 (0.3)
Cholesterol, mmol/l	6.7 (0.5)	6.4 (0.4)*	6.6 (0.5)	6.2 (0.4)#	6.6 (0.5)	5.9 (0.4) Δ	3.6 (0.2)
TG, mmol/l	3.5 (0.6)	3.4 (0.7)*	3.4 (0.7)	3.0 (0.6)#	3.4 (0.7)	2.7 (0.5) Δ	1.5 (0.2)
LDL, mmol/l	4.47 (0.4)	4.1 (0.4)*	4.41 (0.4)	4.1 (0.4)#	4.43 (0.4)	3.7 (0.4) Δ	1.48 (0.2)

BMI, body mass index; TG, triglycerides; LDL, low density lipoprotein; * reliability of the difference before treatment and after treatment in group 1, *p* < 0.05; # reliability of the difference before treatment and after treatment in group 2, *p* < 0.05; Δ reliability of the difference before treatment and after treatment in group 3, *p* < 0.05.

The results showed an improvement in the metabolism of cholesterol after treatment and the ratio of its fractions. Furthermore, there was a decrease in the level of cholesterol and atherogenic lipoproteins (LDL and TG) in all groups. In addition, the level of fasting venous glycemia decreased to reference values in all examined patients during treatment. Moreover, the improvement in the metabolic profile and a decrease in BMI was noted in all patients.

## Discussion

Melatonin reduces hypoxic tissue damage, accelerates ischemic tissue repair, and reduces oxidative stress (Zizi et al., 2010). Our study showed a decrease in the relative expression area of MT receptors in epiphyseal tissues in patients with IC, which may be closely related to the polymorphic metabolic disturbances.

There is an inverse relationship between endogenous melatonin levels and cardiovascular disease (Dominguez-Rodriguez et al., 2010). Nocturnal melatonin synthesis is known to be reduced in patients with coronary heart disease (Altun et al., 2002; Domínguez-Rodríguez et al., 2002), hypertension (Koziróg et al., 2011; Dominguez-Rodriguez et al., 2014), and heart failure (Girotti et al., 2003; Kimak et al., 2014; Dominguez-Rodriguez et al., 2017). The frequency of adverse cardiac events, including myocardial infarction (Domínguez-Rodríguez et al., 2002), sudden cardiac death, and cardiac arrhythmias, increases in the early morning when circulating melatonin levels are significantly lower (Altun et al., 2002). Conclusive studies have been conducted on melatonin levels’ effects and cardiovascular disease incidence (Paulis and Simko, 2007).

Melatonin intake protects the heart in several experimental models of myocardial infarction and myocardial damage (Lochner et al., 2013; Yang et al., 2015). In addition, melatonin infusion (during ischemia and reperfusion or reperfusion alone) reduces premature ventricular contraction and ventricular fibrillation due to occlusion and reopening of the anterior descending coronary artery (Tan et al., 1998).

Also, endogenous and exogenous melatonin plays an important role in hypertension and other vascular pathologies (Grossman et al., 2006; Paulis and Simko, 2007; Możdżan et al., 2014; Simko et al., 2016). In animal studies, continuous exposure to light or pinealectomy followed by melatonin deficiency leads to increased BP and the development of cardiovascular disease (Brown et al., 1991; Iigo et al., 1995).

The results obtained in a sample of young men with polymorbid cardiovascular pathology confirmed the results of the biopsy study. Positive changes in MT excretion in groups 1 and 2 are probably due to the brain’s restructuring of dopaminergic/serotoninergic systems under the influence of antidepressants, tranquilizers, and psychotherapy sessions. In the 3rd group, positive changes can be explained by normalizing the light regime and eliminating the consequences of autonomic nervous systems due to the influence of visual-auditory correction. In all the studied groups, a pronounced pattern of daily excretion of 6-SM was revealed (a decrease in excretion before treatment and the achievement of control values after treatment, *p* < 0.001). Such circadian variations of day/night MT patterns allow us to consider the night excretion of MT as a marker of compensation/decompensation of the central regulatory systems of the autonomic nervous systems in PCVP (Anderson and Maes, 2015).

Analyzing the results of DMBP during the day before treatment, a change of RNP from normal “dipper” to “non-dipper” and “night-peaker” was revealed in all the studied groups. These data can be explained by a change in the state of regulatory systems that are in “compensation” under the influence of vectors of therapeutic measures of the “non-dipper” pattern and the absence of changes in the state of regulatory systems that are in “decompensation” under the influence of vectors of therapeutic measures of the “night-peaker” pattern. With the decrease in MT excretion, vascular wall resistance to endogenous stress mechanisms decreases, microcirculation worsens (lack of stimulation of prostaglandin E2 synthesis, prostacyclin, and other depressors), and in combination with impaired activation of dopaminergic and GABA-ergic processes, the activity of Ca^2+^ channels leads to decompensation of the regulatory mechanisms of hemodynamics (Al-Ghoul et al., 2010; Paredes et al., 2014; Pechanova et al., 2014; Anderson and Maes, 2015).

When analyzing the study results of the CES-D and HADS scales before treatment, data confirming the presence of “moderate clinical anxiety” and “moderate depression” were obtained in all groups. After the treatment, there was a change in the type of DSD from “moderate depression” and “clinical anxiety” to “mild depression” and “subclinical anxiety,” *p* < 0.05. It is important to note that a decrease in the night excretion of MT (night pattern) before treatment significantly correlated with the presence of “moderate depression” (*r* = −0.72) and “clinical anxiety” (*r* = −0.66) in patients with PCVP, while an increase in night excretion after therapeutic measures was associated with a decrease in the severity of anxiety-depressive disorders in all groups of patients (Reiter et al., 2016; Satyanarayanan et al., 2018).

Concomitant anxiety and depressive symptoms have a negative impact on patients with polymorbid cardiovascular pathology (Hare et al., 2014; Tully et al., 2016). However, psychopharmacological therapies may occur metabolic side effects (Kahl et al., 2022), and reduce patients’ compliance by increasing the number of drugs taken (Jin et al., 2008). Recent studies have shown positive results from the use of non-medicinal therapies even among Intensive Care Units patients (Cui et al., 2022), while the use of such techniques among cardiac patients also has a rationale (Cimpean and Drake, 2011; Kahl et al., 2022). At the same time, as our study has shown, psychotherapy and psychophysiological visual and auditory correction may be even more effective in reducing symptoms of anxiety and depression, and restoring melatonin expression in patients with polymorbid cardiovascular pathology.

## Limitations

Our study focuses on assessing 6-SM levels in a male population with polymorbid cardiovascular pathology, and at this stage, the results cannot be extrapolated to female patients, given the possible differences in the hormonal background. However, subsequent work with a similar design in a female or mixed sample could solve this problem. We did not assess the severity of sleep disturbances in patients by means of scales, although these data could further characterize disturbances in circadian rhythms of melatonin extraction.

## Conclusion

Thus, it was revealed that young patients with polymorbid cardiovascular pathology and MS have a lower level of MT excretion at night, which is associated with greater severity of clinical symptoms and a higher risk of death due to ischemic myocardial damage, which was confirmed by the results of a study of biopsy material. We suggest that reduced melatonin levels may increase the risk of cardiovascular disease. However, further studies with an interventional controlled design in large samples are needed to confirm these associations’ primary or secondary origin. Furthermore, similar dynamics were noted when assessing the correlation of the increased level of MT excretion with the severity of anxiety-depressive state and circadian fluctuations in systolic/diastolic BP in patients with the polymorbid pathology under consideration.

The research showed the effectiveness of various approaches in treating patients with PCVP, MS, and DSD. First, however, it is necessary to note the high effectiveness of the standard treatment of PCVP in combination with psychotherapeutic and psychophysiological visual and auditory correction, which contributed to improved metabolic status and carbohydrate and lipid metabolism indices against the background of normalization of circadian rhythm of MT excretion. Furthermore, it is essential to note that the change in laboratory parameters was accompanied by a decrease in the severity of anxiety-depressive disorders and normalization of the circadian BP profile.

## Data availability statement

The original contributions presented in this study are included in the article/supplementary material, further inquiries can be directed to the corresponding author.

## Ethics statement

The studies involving human participants were reviewed and approved by Local Ethic Committee of Kirov Military Medical Academy. The patients/participants provided their written informed consent to participate in this study.

## Author contributions

AP was responsible for the design and execution of the clinical part of the study and the writing of the manuscript. VP was responsible for the design and execution of the immunohistochemical phase of the study and supervision. AT was responsible for calculating the data and preparing the manuscript. DM was responsible for the design and execution of the immunohistochemical phase of the study. DT was responsible for carrying out the immunohistochemical analysis and writing the manuscript. KM was responsible for carrying out the psychiatric part of the study and preparing the manuscript. ESK was responsible for designing and carrying out the psychiatric part of the study. EVK was responsible for preparing the manuscript. AK was responsible for statistical processing and preparation of the manuscript. All authors contributed to the article and approved the submitted version.
